# Development of a Clinical Teaching Evaluation and Feedback Tool for Faculty

**DOI:** 10.5811/westjem.2018.11.39987

**Published:** 2018-12-12

**Authors:** Erin Dehon, Ellen Robertson, Marie Barnard, Jonah Gunalda, Michael Puskarich

**Affiliations:** * University of Mississippi Medical Center, Department of Emergency Medicine, Jackson, Mississippi; † University of Mississippi Medical Center, Department of Neurobiology and Anatomical Sciences, Jackson, Mississippi; ‡ University of Mississippi, Department of Pharmacy, Oxford, Mississippi; § Hennepin County Medical Center, Department of Emergency Medicine, Minneapolis, Minnesota

## Abstract

**Introduction:**

Formative evaluations of clinical teaching for emergency medicine (EM) faculty are limited. The goal of this study was to develop a behaviorally-based tool for evaluating and providing feedback to EM faculty based on their clinical teaching skills during a shift.

**Methods:**

We used a three-phase structured development process. Phase 1 used the nominal group technique with a group of faculty first and then with residents to generate potential evaluation items. Phase 2 included separate focus groups and used a modified Delphi technique with faculty and residents, as well as a group of experts to evaluate the items generated in Phase 1. Following this, residents classified the items into novice, intermediate, and advanced educator skills. Once items were determined for inclusion and subsequently ranked they were built into the tool by the investigators (Phase 3).

**Results:**

The final instrument, the “Faculty Shift Card,” is a behaviorally-anchored evaluation and feedback tool used to facilitate feedback to EM faculty about their teaching skills during a shift. The tool has four domains: teaching clinical decision-making; teaching interpersonal skills; teaching procedural skills; and general teaching strategies. Each domain contains novice, intermediate, and advanced sections with 2–5 concrete examples for each level of performance.

**Conclusion:**

This structured process resulted in a well-grounded and systematically developed evaluation tool for EM faculty that can provide real-time actionable feedback to faculty and support improved clinical teaching.

## INTRODUCTION

Formative evaluations of clinical teaching for emergency medicine (EM) faculty are limited and inadequate.^1^,^2^ Current EM faculty evaluations of teaching are usually based on an entire year and evaluate faculty across a range of teaching, patient care, and research activities using an ordinal scale (e.g., 1 = below expectations, 9 = exceeds expectations).^3^ These summative, end-of-year evaluations of faculty are usually high stakes with linkage to promotion, tenure, awards, and personnel decisions. Summative assessments may be beneficial in determining whether a faculty member is meeting performance standards and can lead to improvements in teaching performance.^4^ However, with summative assessments, faculty are not given the opportunity to integrate feedback into their teaching practice until after receiving results, which doesn’t usually occur until the end of the academic year. Furthermore, summative evaluations tend to focus on broad characteristics of effective teachers vs. specific teaching strategies used to help residents master certain competencies (e.g., communication, procedural skills). Lastly, the results of summative evaluations are often limited in terms of comments with specific feedback.

In contrast to summative evaluations, formative evaluations are typically low stakes and primarily used to provide ongoing feedback for the purpose of performance improvement.^5^ End-of-shift evaluations or daily encounter cards are a commonly used method for providing competency-based feedback to EM residents and medical students about their performance after a shift.^6^ Despite the widespread use of competency-based shift card evaluations for residents and medical students, similar methods have not been applied to faculty. Although one study^2^ describes the feasibility and acceptance of an end-of-shift evaluation for EM faculty, the measure used was not based on well-established teaching competencies nor was it created using scientific standards for instrument development.

EM faculty teaching evaluations and feedback can be improved with the use of proper tools, such as behaviorally-anchored rating scales (BARS). BARS use specific, observable behaviors (i.e., behavioral anchors) that align with competencies at various levels of proficiency. BARS have several benefits compared to traditional rating scales. For one, the use of behavioral anchors helps raters focus on behaviors pertinent to the evaluation and discern what behaviors constitute, for example, “average” vs. “above average” performance.^7^ Furthermore, when raters use a common reference point, inter-rater reliability is improved and evaluation bias is reduced.^8^

Not only can BARS help the resident evaluator but they can also lead to more useful feedback for the faculty member being evaluated.^9^ BARS ensure that faculty are provided with specific and actionable feedback linked to teaching competencies. This would alleviate the frequent problem of residents providing feedback that is vague and nonactionable such as “great teacher” or “not flexible.”^2^ BARS can provide rich feedback to the evaluatee including information about why he or she received a certain rating (e.g., below expectations) and what specific behaviors would lead to improvements in teaching (e.g., exceeds expectations).^10^ We are not aware of any existing measures that use BARS to evaluate and provide EM faculty with feedback about their effectiveness in teaching residents certain skills (e.g., clinical decision-making, patient-centered communication) during a shift. Although the objective structured teaching exercise (OSTE) has been used to evaluate real-time teaching skills of faculty in various specialties, the OSTE was developed for use with standardized teaching encounters and is resource and time intensive.^11^

Thus, there is a need to develop a practical, competency- and behaviorally-based tool for evaluating and providing feedback to EM faculty based on their teaching skills during a shift. We expect that development of a robust evaluation and feedback took will facilitate the provision of specific and actionable feedback and ultimately lead to improvements in clinical teaching. With this in mind, the goal of the present study was to apply the National Institutes of Health (NIH) standards of test development (ie, Patient-Reported Outcome Measurement Information System [PROMIS]) 12 to develop an innovative, semi-quantitative, behaviorally anchored Clinical Teaching Evaluation and Feedback Tool, which will be referred to as a “Faculty Shift Card.”

**Population Health Research Capsule**

What do we already know about this issue?

*While competency-based formative evaluations exist for residents, behaviorally-anchored tools for the assessment of attending bedside teaching are lacking*.

What was the research question?

Can we develop a valid semi-quantitative, behaviorally-anchored clinical teaching evaluation and feedback tool?

What was the major finding of the study?

*A brief, four-item, well-grounded tool was developed to assess major domains relevant to bedside teaching*.

How does this improve population health?

*Standardized assessment using formative evaluations may allow for more actionable feedback in domains related to clinical teaching and benefit medical learners*.

## METHODS

### Study Design

We used processes outlined in the NIH PROMIS standards to develop the Faculty Shift Card. These guidelines are considered the “gold standard” for instrument development. The Faculty Shift Card was developed in three phases: 1) Develop an item bank using focus groups and the nominal group technique (NGT); 2) edit and finalize items using modified Delphi procedure; and 3) finalize the instrument (Table 1).

### Study Setting and Population

We invited a local group of EM educators and EM residents to participate in Phase 1 of this project through two focus groups. A purposive sample included six EM residents and six EM faculty. Resident participants were chosen based on chief status, postgraduate year (PGY) level, and interest in participating. Faculty participants were chosen based on current work in resident education and/or previous teaching awards or nominations. Residents and faculty who participated in the focus groups were compensated for their time. In Phase 2, a national group of seven education experts and a local group of five residents (distinct from Phase 1) were invited to participate via email. We identified experts through networking during the Council of Emergency Medicine Residency Directors (CORD) annual conference and through recommendations from colleagues in CORD. These individuals were not compensated for participating.

### Phase 1: Develop an Initial Item Bank

We conducted two semi-structured focus groups (one with faculty and one with residents) using a modified version of the NGT to develop a comprehensive list of effective teaching behaviors. Following the NGT, each group was presented with specific questions aimed to identify effective and ineffective strategies for clinical teaching in the emergency department (ED). Participants independently generated responses to open-ended questions (Table 2) aimed to identify strategies for teaching skills in the following areas: 1) clinical decision-making; 2) procedures; 3) interpersonal and professional; and 4) multitasking. Each group member privately wrote down his or her response to each question. Then, one-by-one in a round-robin fashion members shared their responses with the group. The group then discussed each idea. After an exhaustive list of potential items was developed, the group anonymously voted “Yes” or “No” on whether or not each item would be able to discriminate among outstanding, average, and poor clinical teachers. If at least two members voted that the item had discriminative value, then the item was maintained for Phase 2.

The focus group co-facilitators (Erin Dehon and Ellen Robertson) developed a list of the items identified during the focus groups. We combined similar items listed by the faculty and resident groups. The results were collated and used to develop a survey for Phase 2.

### Phase 2: Edit and Finalize Items Using Modified Delphi Procedure

#### Delphi Round 1

An anonymous survey of the items developed in Phase 1 asked resident and faculty group participants to review and rate each item on a scale from 1–4 (1 = not important, 2 = somewhat important, 3 = moderately important, 4 = extremely important). We used the responses to calculate a content validity index (CVI) to determine which items to retain.^13^ The CVI for each item is the proportion of individuals who rated the item as 3 or 4 (extremely or moderately important) vs. 1 or 2 (somewhat or not important). For example, if five out of 15 reviewers rated an item as a 3 or 4, then the CVI would be 5/15 = 0.33. As recommended in the literature, items with a CVI less than 0.83 were dropped.^13^

#### Delphi Round 2

In Phase 2 we solicited feedback via email from a select group of six expert educators in EM residency training about the items generated in Phase 1. Specifically, we invited experts to participate in an anonymous survey to review each potential item and rate each item’s level of importance in terms of helping residents develop competency in the following: 1) clinical decision-making, 2) procedural skills, 3) multitasking, and 4) interpersonal communication. Items were rated on a three-point scale (1 = extremely important, 2 = somewhat important, 3 = not important). Experts were also asked to list any additional items that they felt were important but missing. If the majority of experts (four or more) rated an item as extremely important, it was maintained for round 3. Items rated by only one or two experts as extremely important were dropped. Items rated by three experts as extremely important and three experts as somewhat or not important were evaluated by the study authors for potential deletion.

#### Delphi Rounds 3 and 4

The goal of these rounds was for a group of residents (separate from those in Phase 1) to reach consensus about the category of expertise for each of the teaching behaviors previously identified. First, they were sent a survey and asked to classify the identified teaching strategies into one of three options for level of expertise: novice — everyone does this; intermediate — majority of good teachers do this; and advanced — only the top 25% of teachers do this. The survey responses were then returned to all participants and they were asked once again to categorize the teaching behaviors, taking into account everyone else’s responses. This round was repeated once for the items that did not reach consensus. Consensus was defined as at least four of the five residents agreeing on the classification level.

### Phase 3: Finalizing the Instrument

In the final phase we conducted a literature review to ensure no key constructs were missing. Then, we created a prototype of the Faculty Shift Card and invited residents from the previous phase to provide feedback on it.

## RESULTS

### Phase 1: Develop an Initial Item Bank

The faculty focus group included six faculty members including the program director, two associate program directors, and two other faculty who were recipients of the yearly teaching award. The resident focus group included six residents comprised of three chief residents and one resident from each of the other classes (PGY1-PGY3). During the NGT session, resident participants identified a total of 52 teaching behaviors that are able to discriminate among outstanding, average, and poor clinical teachers. Faculty participating in the NGT session identified a total of 52 teaching behaviors deemed as having discriminative value.

Two study authors aggregated the content of the 52 resident and 52 faculty responses. There were 22 unique resident responses and 23 unique faculty responses. We pared down the remaining 59 items to 16 items based on redundancy between groups or overlap with other items. The resulting 61 items were organized based on one of the four domains: teaching clinical decision-making (n=19); teaching interpersonal skills (n = 12); teaching procedural skills (n = 10); and teaching task-switching (n = 9). General items that did not apply to any of these specific teaching domains were grouped together and labeled as general teaching strategies (n =11) (e.g., showing an interest in teaching, being available). The full list of items and the Delphi process are in the Supplemental Table.

### Phase 2: Modified Delphi

#### Delphi-Round 1: Resident and Faculty Review

All 12 faculty and residents who participated in the focus groups from Phase 1 completed the survey for the first round. This round began with 61 preliminary items. Participants rated the majority of these items as extremely or moderately important. In this round, 10 items had CVIs less than 0.83 and were deleted, leaving 51 items.

#### Delphi-Round 2: Expert Review

Of the seven experts invited to participate, six agreed and completed the survey in full. All experts were emergency physicians working in an academic medical center with experience teaching EM residents. All experts were members of CORD and included a program director, simulation director, ultrasound director, and faculty members with publications in medical education. Delphi round 2 began with 51 preliminary items. The six expert participants did not rate any of the items as “not important.” In this round, nine items were dropped due to low ratings of importance by experts (≤3). The experts also added two items to the domain teaching clinical decision-making, which resulted in a list of 44 items.

#### Delphi-Rounds 3 and 4: Item Classification

The five residents who participated in these rounds included three chief residents, a PGY-2 resident, and a PGY-4 resident. Delphi round 3 began with 44 items that residents were asked to classify into categories. After round 1, residents reached consensus on 24 of the 44 items. After round 2, 39 of 44 items reached consensus. The five items that did not reach consensus were dropped (Table 3).

### Phase 3: Finalize the Instrument

**Figure 1:**
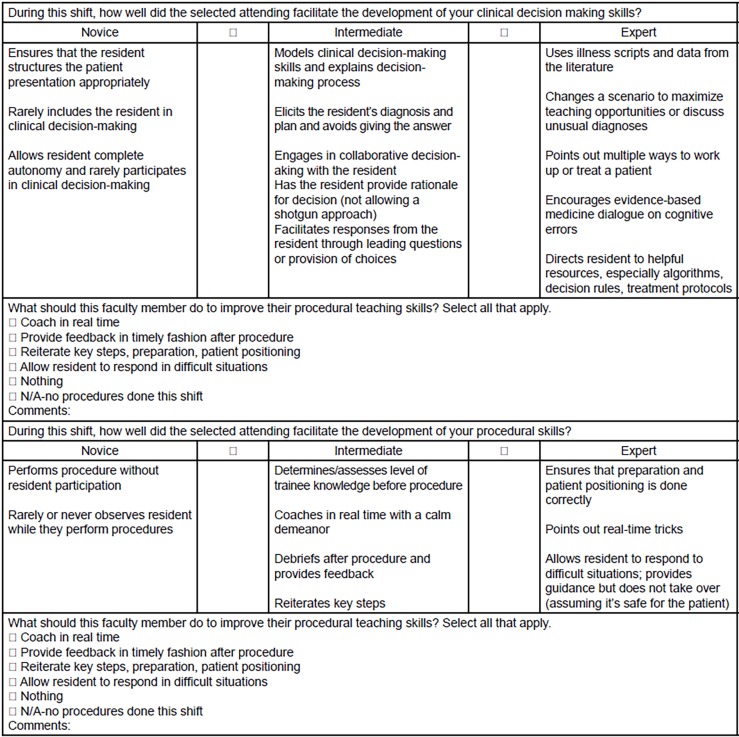
Faculty shift card 1.

**Figure 2:**
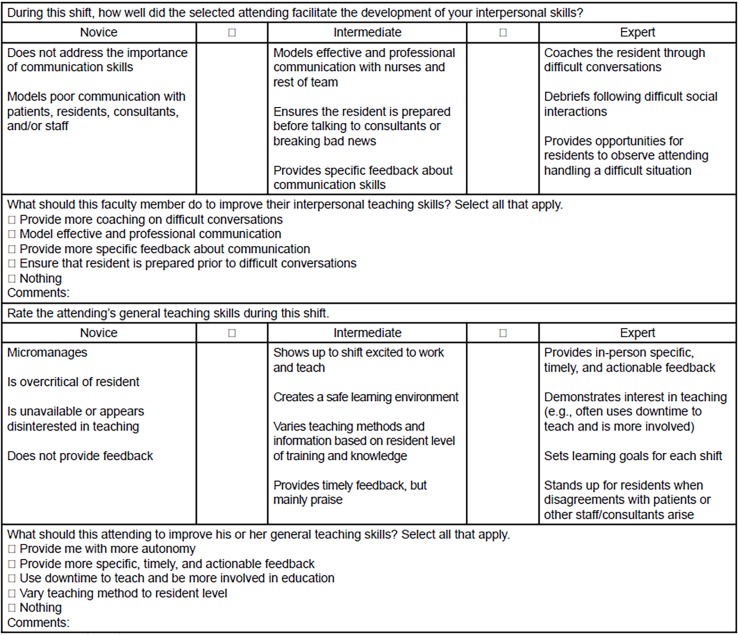
Faculty shift card 2.

After 39 important teaching behaviors were established and categorized by the consensus groups, we conducted a thorough literature review focused on identifying the behaviors and characteristics of effective clinical teaching in the ED, the features of effective written feedback for faculty, and existing validated clinical teaching instruments (including those designated for other specialties). This review helped ensure that no items were missing and informed fine-tuning of the final instrument (Figures 1 and 2). The items on the instrument were found to be in line with the existing literature on teaching competencies in graduate medical education,^14^ as well as with EM faculty strategies for good teaching.^15^ Fine-tuning involved combining items on the Faculty Shift Card, as well as rephrasing several positive items to reflect less-desirable behaviors to place in the novice category (e.g., “providing autonomy” to “micromanages”). Items were also edited to ensure use of concrete behavior anchors to facilitate more consistent and actionable feedback across residents of varied program years and educational needs.

We were able to incorporate all of the teaching behaviors identified as important in the previous stages into a brief four-item tool. Each of the four items focused on a specific domain: 1) clinical decision-making skills; 2) procedural skills, 3) communication skills, and 4) general teaching strategies. As can be seen in Figures 1 and 2, the task-switching domain was dropped from the final Faculty Shift Card. The investigators decided to drop this domain since faculty, residents, and a literature search did not lead to identifying clearly defined strategies for teaching task-switching.

In response to suggestions from residents, an optional comment box was added and we divided the items into two, two-item shift cards to shorten them. Shift Card 1 includes clinical decision-making and procedural knowledge, and Shift Card 2 includes interpersonal skills and general teaching.

## DISCUSSION

We developed two, two-item faculty shift cards using the NIH PROMIS standards of test development. This article describes a systematic and iterative process of developing an innovative Faculty Shift Card. Ultimately, the aim of this tool is to improve clinical teaching in EM by providing EM attendings with more frequent specific and actionable feedback about their clinical teaching practices during a shift.

To ensure content validity, the Faculty Shift Card was developed systematically through a thorough literature review and input from residents, faculty, experts using qualitative and survey methodology. We used the NGT and modified Delphi method to obtain opinions from residents, faculty, and expert educators about important strategies that faculty use to teach certain fundamental skills: clinical decision-making; procedural; interpersonal; and task-switching. Overall, resident and faculty perceptions of effective clinical teaching strategies were remarkably similar. It is worth noting that regardless of the specific skill being taught, all respondents emphasized the importance of the core characteristics of effective teachers, which included being available, supportive and approachable, and demonstrating an interest in teaching. This led to the development of an item focused on general teaching strategies.

Given that both faculty and residents had a difficult time identifying clear strategies for teaching task-switching, we excluded this item from the final tool. Although task-switching is a core competency that residents are expected to develop throughout their training, effective practices for teaching task-switching are lacking.^16^ Role modeling was noted as the main method of teaching task-switching, but it was not explicitly clear how role modeling was being used to teach how to manage multiple patients and tasks. Before we can properly evaluate faculty’s ability to teach task-switching, we need better-defined strategies to effectively teach this skill.

The Faculty Shift Card has several advantages. It is a short yet comprehensive tool for evaluating and providing formative feedback to EM faculty aiming to improve their clinical teaching skills. This four-item tool incorporated all 38 teaching behaviors identified as essential to effective clinical teaching in the ED. It was divided into two, two-item shift cards after receiving feedback from residents that the four-item tool may be too time-consuming. The tool could also be easily adapted to four one-item shift cards. The brevity of this tool lends itself to routine use in the ED setting. Furthermore, each item on the shift card provides a list of specific and actionable feedback that residents can select to give faculty, thereby resolving the problem of residents’ tendency to provide faculty with vague feedback.^2^

## LIMITATIONS

There are several limitations to consider. First, the shift card was developed at a single institution. Thus, the proposed set of criteria may be influenced based on local priorities and culture. However, we mitigated these limitations by the engagement of national experts and a thorough literature review. Evaluation of the local face and construct validities of the instrument should be considered prior to its use in other settings. Additionally, members of the focus groups were chosen based on factors such as chief status, engagement in resident education, faculty teaching award recipients, and overall interest. Although we were able to include residents of all PGY levels in Phase 1 of the development process, Delphi rounds 3 and 4 did not include representation from the intern class due to a lack of volunteers from that class. Without reliable assessment tools already developed, these persons may or may not represent the most effective teachers or the most insightful in identifying effective teaching behaviors. Further testing of the instrument, specifically to assess whether the instrument is effective in discriminating between effective and ineffective clinical teachers and whether actionable feedback leads to changes in faculty teaching behaviors, is indicated. Nevertheless, the approach to the development and application of a valid instrument for this purpose does have some novelty.

## CONCLUSION

Using a modified Delphi approach with local departmental leaders in education with input from national experts, we developed a semi-quantitative, behaviorally-anchored clinical teaching evaluation and feedback tool, the Faculty Shift Card, which can provide real-time actionable feedback to faculty and support improved clinical teaching. Testing the efficacy of the tool to affect faculty teaching behaviors is indicated.

## Supplementary Material



## Figures and Tables

**Table 1:** Phases of faculty shift card development.

Phases of development	Actions
Phase 1 – develop an initial item bank	Conducted faculty and resident focus groups using the nominal group technique
	Developed preliminary item list by aggregating faculty and resident items and removing redundant items
Phase 2 – finalize items using modified Delphi method	Conducted four Delphi rounds:
	Delphi Round 1: Content validity index of initial resident and faculty participants used to determine item inclusion
	Delphi Round 2: Emergency medicine education experts surveyed for item inclusion
	Delphi Round 3: Residents surveyed to classify items into novice/intermediate/advanced
	Delphi Round 4: Classification repeated for non-consensus items from round 3
Phase 3 – finalize the instrument	Conducted literature review to ensure no key constructs were missing
	Refined final instrument

**Table 2:** Focus group interview questions.

What are effective teaching strategies that faculty use during shifts that help you master clinical decision-making (e.g., selecting the most appropriate diagnostic test, developing a differential diagnosis, choosing the most appropriate treatment, practicing evidence-based medicine)? What are effective teaching strategies that faculty use during shifts that help you master procedural knowledge/skills (e.g., ultrasound, airway management, performing a history and physical examination)? What are effective teaching strategies that faculty use during shifts that help you master interpersonal skills (communicating effectively with nurses, patients, families, breaking bad news, etc.)? Task-switching is a core skill in emergency medicine — What are the best strategies for teaching task-switching and how to manage multiple patients? What are ineffective teaching strategies that faculty use during shifts?

* The faculty group was asked a slightly modified version of the same questions.

**Table 3:** Stages of the nominal group technique and modified Delphi process used to develop the faculty shift card.

Teaching domains for instrument	Item pool development(number of items remaining at conclusion of each round)			Classification of items by level of teaching expertise (number of items consensus reached at the conclusion of each round)		
	
	Initial item set	Delphi round 1	Delphi round 2	Delphi round 3	Delphi round 4	Consensus not reached (items dropped)
Clinical decision making	19	12	*12	9	11	1
Task-switching	9	6	3	0	3	0
Communication	12	12	10	6	8	2
Procedural	10	10	8	2	7	1
General teaching	11	11	11	7	10	1
Total items	61	51	44	24	39	5

* 2 removed, 2 added.
