# Supplementary material for: Development of a Clinical Teaching Evaluation and Feedback Tool for Faculty
Source: West J Emerg Med. 2018 Dec 12;20(1):50–7. doi: 10.5811/westjem.2018.11.39987 (PMC6324693; doi:10.5811/westjem.2018.11.39987)
Supplement: Supplementary file 1 [file wjem-20-50-s001.docx]

Table

| Phase 1: Items Identified During Nominal Group Technique | Delphi: Round 1 | Delphi Round 2 | Delphi Round 3 | | | Delphi Round 4 | | |  |
| --- | --- | --- | --- | --- | --- | --- | --- | --- | --- |
| How important is it for faculty to use the following strategies when helping residents master…. | Residents (n=6) and Faculty (n=6) CVI | # Experts (n=6) rated as Very Important  Expert CVI | N | I | E | N | I | E |  |
| **CLINICAL DECISION MAKING?** | | | | | | | | |  |
| 1. Explain their decision making process | 1.00 | 5 | 0 | 1 | **4** |  |  |  | E |
| 1. Ask questions to lead the trainee to the solutions (versus providing the answer) | 1.00 | 5 | 0 | 4 | 1 |  |  |  | I |
| 1. Point out multiple solutions (multiple ways to work up or treat a patient) | 1.00 | 4 | 0 | 1 | 4 |  |  |  | I |
| 1. Have the resident to provide rationale for decision (not allowing a shotgun approach) | 0.92 | 6 | 1 | 2 | 2 |  | 4 | 1 | I |
| 1. Creates a safe learning environment/ Does NOT criticize resident decisions | 0.92 | 4 | 1 | 4 | 0 |  |  |  | I |
| 1. Not intimidating, remaining calm when things go wrong | 1.00 | 4 | 1 | 4 | 0 |  |  |  | I |
| 1. Gear questions and teaching points toward level of training | 0.92 | 5 | 1 | 3 | 1 |  | 4 | 1 | I |
| 1. Model clinical skills (such as important questions to ask during H & P) | 0.83 | 4 | 1 | 3 | 1 |  | 3 | 2 | NC* |
| 1. Change the scenario to maximize teaching points | 0.83 | 3 | 0 | 0 | 5 |  |  |  | E |
| 1. Take opportunities to bring up "What ifs?" - take time to talk about unusual diagnosis | 0.92 | 3 | 0 | 0 | 5 |  |  |  | E |
| 1. Have the resident work through differential diagnosis with attending | 0.92 | 1* |  |  |  |  |  |  |  |
| 1. Allow residents autonomy to make decisions and learn from mistakes | 0.92 | 1* |  |  |  |  |  |  |  |
| 1. Teach the resident how to present a patient | 0.67* |  |  |  |  |  |  |  |  |
| 1. Work with the resident to establish outcome goals for the patient prior to treatment | 0.67* |  |  |  |  |  |  |  |  |
| 1. Provide case examples from their own experience | 0.58* |  |  |  |  |  |  |  |  |
| 1. Provide residents with opportunities to observe their clinical skills and patient interactions | 0.75* |  |  |  |  |  |  |  |  |
| 1. Help with management of cases and patient load | 0.75* |  |  |  |  |  |  |  |  |
| 1. Direct and encourage use of helpful resources (algorithms, decision rules, tx protocols) | 0.75* |  |  |  |  |  |  |  |  |
| 1. Revisit patient with resident and follow-up | 0.75* |  |  |  |  |  |  |  |  |
| 1. Encourage Evidence based dialogue on cognitive errors (item added by experts) |  |  | 0 | 0 | 5 |  |  |  | E |
| 1. Use illness scripts and data from the literature (item added by experts) |  |  | 0 | 1 | 4 |  |  |  | E |
| **TASK SWITCHING?** | | | | | | | | |  |
| 1. Teach residents how to anticipate what might need to be done | 0.92 | 5 | 0 | 2 | 3 |  | 1 | 4 | E |
| 1. Model how to delegate and track multiple patients | 0.83 | 5 | 1 | 1 | 3 |  |  | 5 | E |
| 1. Model how to deal with interruptions | 0.83 | 4 | 0 | 2 | 3 |  |  | 5 | E |
| 1. Teach residents how to prioritize tasks (when to order tests and see patients) | 1.00 | 3* |  |  |  |  |  |  |  |
| 1. Model how to effectively balance patients and charting | 1.00 | 2* |  |  |  |  |  |  |  |
| 1. Give advice on how to effectively balance patients and charting | 0.83 | 2* |  |  |  |  |  |  |  |
| 1. Ask residents to provide updates on patients | 0.75 |  |  |  |  |  |  |  |  |
| 1. Give residents structured time management strategies | 0.58 |  |  |  |  |  |  |  |  |
| 1. Point out, critiquing poor time management | 0.67 |  |  |  |  |  |  |  |  |
| **INTERPERSONAL SKILLS?** | | | | | | | | |  |
| 1. Be available and support the resident in tough situations | 1.00 | 5 | 1 | 3 | 1 |  | 4 | 1 | I |
| 1. Model and stress importance of effective and professional communication with nurses and rest of team | 1.00 | 5 | 2 | 1 | 2 | 1 |  | 4 | E |
| 1. Provide opportunities for residents to observe how an attending handles a difficult situation (breaking bad news, difficult consultant) | 0.92 | 5 | 0 | 1 | 4 |  |  |  | E |
| 1. Provide feedback to residents about their interaction with patients, families, and healthcare team | 0.83 | 5 | 0 | 1 | 4 |  |  |  | E |
| 1. Debrief resident following difficult social situations | 0.92 | 6 | 0 | 1 | 4 |  |  |  | E |
| 1. Review the reason for consult before calling the consultant | 0.83 | 4 | 2 | 1 | 2 | 1 | 1 | 3 | NC* |
| 1. Model enthusiasm and positivity | 1.00 | 4 | 0 | 4 | 1 |  |  |  | I |
| 1. Coach the resident through difficult conversations | 0.83 | 6 | 0 | 1 | 4 |  |  |  | E |
| 1. Treat breaking bad news as a procedure (have the resident rehearse ahead and give feedback) | 0.83 | 3 | 0 | 0 | 5 |  |  |  | E |
| 1. Have residents discharge the patient, remind them to wrap up with patient, ask patient if they have any questions | 0.83 | 3 | 2 | 0 | 3 | 2 | 1 | 2 | NC* |
| 1. Give examples on how to break bad news | 1.00 | 2* |  |  |  |  |  |  |  |
| 1. Give advice on how to communicate effectively with patients, staff, and consultants | 1.00 | 2* |  |  |  |  |  |  |  |
| **PROCEDURAL?** | | | | | | | | |  |
| 1. Assess level of trainee confidence and competence before doing a procedure/patient encounter | 0.92 | 5 | 1 | 3 | 1 |  | 4 | 1 | I |
| 1. Be available and Support the resident in tough situations | 1.00 | 5 | 2 | 3 | 0 |  | 4 | 1 | I |
| 1. For inexperienced residents ask them to verbally walk through the procedure first | 0.83 | 5 | 2 | 0 | 3 |  |  | 5 | E |
| 1. Coach in real time with a calm demeanor | 0.83 | 5 | 0 | 5 | 0 |  |  |  | I |
| 1. Reiterate key steps | 0.83 | 4 | 2 | 0 | 3 |  |  | 5 | E |
| 1. Allow trainee to respond to difficult situations/procedures, guide but do not take over (assuming it's safe for the patient) | 0.92 | 4 | 0 | 2 | 3 |  | 2 | 3 | NC* |
| 1. Have the resident teach the procedure | 0.83 | 4 | 0 | 1 | 4 |  |  |  | E |
| 1. Stress kit preparation and patient positioning—is it all laid out correctly | 1.00 | 3 | 0 | 2 | 3 |  |  | 5 | E |
| 1. Teach real time tricks | 0.92 | 3* |  |  |  |  |  |  |  |
| 1. Ask resident the indications/contraindications or potential complications of procedure | 0.92 | 3* |  |  |  |  |  |  |  |
| **GENERAL?** | | | | | | | | |  |
| 1. Show up to shift with a positive attitude | 1.00 | 6 | 1 | 3 | 1 |  | 4 | 1 | I |
| 1. Create a safe learning environment | 1.00 | 5 | 1 | 4 | 0 |  |  |  | I |
| 1. Provide in-person specific, timely, and actionable feedback | 0.83 | 6 | 0 | 2 | 3 |  |  | 5 | E |
| 1. Be available (not checking email or facebook) | 1.00 | 6 | 0 | 4 | 1 |  |  |  | I |
| 1. Provide Support- Stand up for residents when disagreements with patients or other staff arise | 1.00 | 6 | 0 | 0 | 5 |  |  |  | E |
| 1. Demonstrate interest in teaching (uses downtime to teach) | 1.00 | 6 | 0 | 0 | 5 |  |  |  | E |
| 1. Provide autonomy and NOT micromanage residents | 1.00 | 6 | 0 | 2 | 3 |  | 2 | 3 | NC* |
| 1. Be Approachable- Respond to questions from residents without being aggressive or getting offended that they asked why | 1.00 | 6 | 0 | 5 | 0 |  |  |  | I |
| 1. NOT criticize residents | 0.83 | 4 | 1 | 3 | 1 |  | 4 | 1 | I |
| 1. NOT manage residents' patients for them | 0.83 | 4 | 0 | 5 | 0 |  |  |  | I |
| 1. Provide Constructive Feedback Privately | 0.83 | 5 | 0 | 4 | 1 |  |  |  | I |

Note : N = Novice, I = Intermediate, E = Expert; *Item dropped
